# Supplementary material for: Dog Domestication Strongly Relied on Translation Regulation According to Differential Gene Expression Analysis
Source: Animals (Basel). 2024 Sep 12;14(18):2655. doi: 10.3390/ani14182655 (PMC11428534; doi:10.3390/ani14182655)
Supplement: Supplementary file 1 [file animals-14-02655-s001.zip › consent_form_EN.pdf]

Document identification number:

Consent Form

– Consent to blood sampling from your dog –

I, the undersigned .... (hereinafter the Owner) hereby declare that I give my full consent to the Department of Ethology, ELTE TTK (Budapest 1117, Pázmány Péter sétány 1/C, hereinafter referred to as the Department) to take blood from my dog. My contribution is voluntary and without financial reward, and is intended to support the development of scientific research, in particular veterinary science, molecular biology, genetics and ethology, and to promote education and medicine.

I declare that I have ownership and/or proprietary rights of the following animal:

- o Name of the dog: ……………………………… o Breed: ………………………………….
- o Sex: ☐ bitch ☐ dog o Neutered status: ☐ yes ☐ no
- o Date of birth: ……………………………
- o Is the date of birth accurate? ☐ yes ☐ no
- o Transponder ID: ☐☐☐ ☐☐☐☐ ☐☐☐☐ ☐☐☐☐

I certify that the above named dog is healthy at the time of the blood sampling, has not suffered from any chronic illness in his life and has not taken any medications in 2 weeks prior to the sampling. The dog has no symptoms of epilepsy and has not been diagnosed with cancer.

Comment: ....

By signing and submitting this consent form, I agree that the Department may use the obtained samples in full compliance with the above mentioned purposes, dispose them and/or use them for research purposes.

<u>Contact Form</u>:

☐ Yes, I agree that, if necessary, I may be contacted by the Department's staff to ask questions about my dog's behaviour, habits and veterinary history.

E-mail: ....

Phone number: ....

☐ I do not agree to be contacted.

I confirm that I have read the consent form and I understand and accept its terms and conditions.

Dated: ………………………… , ………. (YYYY) ……. (MM) ……. (DD).

....

Signature of owner
