# Supplementary material for: Dog Domestication Strongly Relied on Translation Regulation According to Differential Gene Expression Analysis
Source: Animals (Basel). 2024 Sep 12;14(18):2655. doi: 10.3390/ani14182655 (PMC11428534; doi:10.3390/ani14182655)
Supplement: Supplementary file 1 [file animals-14-02655-s001.zip › consent_form_HUN.pdf]

Iktatási szám: …………………………...

Hozzájáruló nyilatkozat

– kutya vérvételéhez való hozzájárulás –

Alulírott ……………………………………………………………..(a továbbiakban: Tulajdonos) jelen nyilatkozatomban kijelentem, hogy hozzájárulok ahhoz, hogy az ELTE TTK Etológia Tanszék (Budapest 1117, Pázmány Péter sétány 1/C, a továbbiakban: Tanszék) számára vért vegyenek a kutyámtól. Hozzájárulásom önkéntes és anyagi ellenszolgáltatás nélküli, célja a tudományos kutatás, különösen az állatorvos-tudomány, a molekuláris biológia, genetika és az etológia fejlődésének támogatása, az oktatás és gyógyítás segítése.

Kijelentem, hogy az alábbi állat felett tulajdonosi és/vagy rendelkezési jogom van:

- A kutya neve: ………………………………… o Fajtája: ………………………………….
- Neme: ☐ szuka ☐ kan o Ivartalanított-e: ☐ igen ☐ nem
- Születési ideje: ……………………………
- Becsült-e a születési dátum? ☐ igen ☐ nem
- Mikrochip száma: ☐☐☐ ☐☐☐☐ ☐☐☐☐ ☐☐☐☐

Kijelentem, hogy a fent nevezett kutya a vérvétel pillanatában egészséges, élete során nem szenvedett semmilyen krónikus betegségben, a vérvételt megelőző 2 hétben nem szedett gyógyszert. A kutyának nincsenek epilepsziára utaló tünetei, nem diagnosztizáltak nála rákos megbetegedést.

Megjegyzés:……………………………………………………………………………………...

Nyilatkozatommal hozzájárulok, hogy a Tanszék a fenti célokkal összhangban rendelkezzen az átvett mintákkal, azokat megőrizze, és kutatási célból felhasználja.

<u>Kapcsolatfelvételi nyilatkozat</u>:

☐ Igen, hozzájárulok, hogy amennyiben szükséges, a Tanszék munkatársai felvehessék velem a kapcsolatot azzal a céllal, hogy kérdéseket tegyenek fel a kutyám viselkedéséről, szokásairól és az állatorvosi kórelőzményekről.

email-cím: ………………………………………………………….…..

telefonszám: …………………………………………………..…..

☐ Nem járulok hozzá a kapcsolatfelvételhez.

A hozzájáruló nyilatkozatot elolvastam, a bennük foglaltakat megértettem és elfogadom.

Kelt ….………………………. , ……..… év ……. hó ……. nap.

……………………………………………

Tulajdonos aláírása
