# Supplementary material for: Dog Domestication Strongly Relied on Translation Regulation According to Differential Gene Expression Analysis
Source: Animals (Basel). 2024 Sep 12;14(18):2655. doi: 10.3390/ani14182655 (PMC11428534; doi:10.3390/ani14182655)
Supplement: Supplementary file 1 [file animals-14-02655-s001.zip › supplementary_materials_20240909.pdf]

Supplementary materials

Manuscript title: Dog domestication strongly relied on translation regulation according to differential gene expression analysis

Authors
David Jonas[1,2], Kitti Tatrai[1,3], Sara Sandor[1], Balazs Egyed[3], Eniko Kubinyi[1,2,4]

Affiliations
1. Department of Ethology, ELTE Eötvös Loránd University, 1117 – Budapest, Hungary
2. MTA-ELTE Lendület "Momentum" Companion Animal Research Group, 1117 – Budapest, Hungary
3. Department of Genetics, ELTE Eötvös Loránd University, 1117 – Budapest, Hungary
4. ELTE NAP Canine Brain Research Group, 1117 – Budapest, Hungary
Corresponding author(s): David Jonas (jonas.david@ttk.elte.hu)

Re-analysis of the Asian samples of Yang et al. [11] – Supplementary methods
In this analysis we aimed to reproduce the results published by Yang et al. in 2018. To do so, we used only the Asian samples and the CanFam 3.1 reference genome with genome annotations from Ensembl v. 98 [15]. We also excluded the continent ID from the model as all animals were from Asia.
In accordance with the applied analysis workflow, we defined an *expressed gene* as a gene that contained minimum ten reads in at least two individual samples. The minimum number of samples was reduced to two as compared to the main study, where this parameter was eleven; this was necessary as the smallest cluster included only two samples in this reduced experiment (n=2 in the dog cluster). With this definition, we found 12435 expressed genes in our samples.

Supplementary data S1: the complete list of significant gene ontology terms from the main analysis.

Supplementary tables

Table S1 Summary table of the experimental laboratory and sequencing methods of the four different datasets used in the analysis. In case of Yang et al. and Jónás et al., the bioinformatic methods are also presented.

Experimental step	Yang et al. [11]	Charruau et al. [14]	Liu et al. [13]	Jónás et al. [12]
		Experimental laboratory		
Biological sample	Whole blood	Whole blood	Whole blood after anesthesia	Whole blood
Blood preservation	RNAprotect Animal Blood Tubes (QIAGEN)	PAXgene Blood RNA tubes (PreAnalytiX, Qiagen	RNAprotect® Animal Blood Tubes (QIAGEN)	DNA/RNABlood Collection Tubes (Zymo Research).
Blood storage	-80 °C	-80 °C	Liquid nitrogen	-20 °C
RNA extraction	RNeasy Protect Animal Blood Kit (QIAGEN)	PAXgene Blood RNA kit (PreAnalytiX, Qiagen)	RNeasy® Protect Animal Blood Kit (QIAGEN)	Quick-DNA/RNA Blood Tube kits (Zymo Research)
RNA storage	Not published	Not published	Not published	-80C
RNA treatment	DNase	Not published	Not published	DNase
RIN[1]	8.8-8.9	7<	8.0-9.2	8.5-10.0
Enrichment	polyA capture	Globin-Zero kit (Illumina), purified with a modified Qiagen RNeasy MinElute (Qiagen) OR ethanol precipitation	TruSeq SR Cluster Kit v3-cBot-HS (Illumia)	polyA capture
Library preparation	Illumina Tru-Seq RAN Low Sample (LS) protoco	Epicentre (ScriptSeq v2 Library Prep kit, Illumina, Madison, WI) with ScriptSeq Index PCR primers (Epicentre, Illumina,	NEBNext® Ultra™ RNA Library Prep Kit for Illumina®	TruSeq® Stranded mRNA Library preparation kit (Illumina, CA, USA)
Sequencing	Illumina HiSeq 2000, 5GB, 125 PE	HiSeq2000, Single-end 100 bp sequencing	Illumina Hiseq 2500 platform and 125 bp paired-end reads	Novaseq 6000, read no: 42M, 150 PE
		Bioinformatics analysis		
Reference genome	CanFam 3.1	The exact methodology of the implemented scientific research in this study is not relevant for the current research.	The exact methodology of the implemented scientific research in this study is not relevant for the current research.	ROS Cfam 1.0
Sample size	3 wolves, 2 dogs			11 wolves, 12 dogs
Raw data quality control	Software not published			Software: FastQC

Adapter and raw data quality trimming	Software not published; reads with more than 10% Ns removed; reads containing adapters and low quality reads removed			cutadapt: minimum read length 50 bp; trailing polyG and adapter sequences removed
Alignment	Software: Tophat, default parameters			Software: HISAT2, default parameters except for the --dta option
Transcript assembly	Software: Cufflinks			Not implemented
Hemoglobin removal	Removal not implemented			Software: picard, samtools and in house scripts
Count matrix	Software: HTSeq R package; FPKM normalization implemented			Count matrix created with the Rsubread R package; multi-mapped reads not counted, paired-end option set
Differential gene expression analysis	DESeq2 and edgeR; $q-value < 0.05$ and $\|log_2fold\ change\| > 1$ thresholds implemented[2]			Software: DESeq2<br>Continent effect included as a confounding factor in the design formula; genes considered *expressed* if they were sequenced in minimum 11 animals (minimum 10 read/animal)
GO analysis	As implemented in the clusterProfiler R package			Software: pantherDB on-line tool<br>Reference gene set: the expressed set of genes identified in this study; correction for multiple testing: based on FDR[3]
WGCNA[4]	Not implemented			As implemented in the WGCNA R package
KEGG analysis	Software: Kobas			Not implemented

1 RIN – RNA integrity number; 2 the q-value threshold was used only in the *subset()* function of DESeq2 and was not used in the *results()* function.; 3 FDR – false discovery rate;4: WGCNA – weighted correlation network analysis

Table S2 Hemoglobin-related genes from Harrington et al. [10] and their canine orthologs together with genomic information about the genes. The dashed line separates the active and archived canine genes in Ensembl's annotation database.

Chr ID	Gene start	Gene end	Strand	Dog gene ID	Human gene ID	Human gene abbreviation	Human gene name	Gene type	Ortholog type	Dog gene's status
6	40675294	40676260	Reverse	ENSCAFG00845001143	ENSG00000130656	HBZ	hemoglobin subunit zeta	Protein coding	One to many	Active
6	40685038	40686021	Reverse	ENSCAFG00845001152						Active
6	40577773	40672545	Reverse	ENSCAFG00845000903	ENSG00000188536	HBA2	hemoglobin subunit alpha 2	Protein coding	One to many	Active
NA	NA	NA	NA	NA	ENSG00000196565	HBG2	hemoglobin subunit gamma 2	Protein coding	NA	NA
6	40577773	40672545	Reverse	ENSCAFG00845000903	ENSG00000206172	HBA1	hemoglobin subunit alpha 1	Protein coding	One to many	Active
6	40674308	40675002	Reverse	ENSCAFG00845001118	ENSG00000206177	HBM	hemoglobin subunit mu	Protein coding	One to one	Active
NA	NA	NA	NA	NA	ENSG00000213934	HBG1	hemoglobin subunit gamma 1	Protein coding	NA	NA
NA	NA	NA	NA	NA	ENSG00000223609	HBD	hemoglobin subunit delta	Protein coding	NA	NA
NA	NA	NA	NA	NA	ENSG00000244734	HBB	hemoglobin subunit beta	Protein coding	NA	NA

Table S3 Biotype classification of the significantly differentially expressed genes. Only the number of genes per biotype is shown; the biotype descriptions are quoted from Ensembl's website.

Ensembl biotype	Biotype description[1]	All expressed gene count	Differentially expressed gene count	Notes
Protein coding	Gene/transcript that contains an open reading frame (ORF)	11324	1502	
Pseudogene	A gene that has homology to known protein-coding genes but contains a frameshift and/or stop codon(s) which disrupts the ORF. Thought to have arisen through duplication followed by loss of function	49	10	
Processed pseudogene	Pseudogene that lacks introns and is thought to arise from reverse transcription of mRNA followed by reinsertion of DNA into the genome	4	3	A subset of the *pseudogene* biotype
lncRNA	A non-coding gene/transcript >200bp in length	578	47	A subset of the *Processed transcript* biotype
IG C gene	Constant chain immunoglobulin gene that undergoes somatic recombination before transcription	7	2	A subset of the *IG gene* biotype
IG V gene	Variable chain immunoglobulin gene that undergoes somatic recombination before transcription	8	3	A subset of the *IG gene* biotype
miRNA	A small RNA (~22bp) that silences the expression of target mRNA	16	7	A subset of the *ncRNA* biotype
snRNA	Small RNA molecules that are found in the cell nucleus and are involved in the processing of pre messenger RNAs	2	0	A subset of the *ncRNA* biotype
snoRNA	Small RNA molecules that are found in the cell nucleolus and are involved in the post-transcriptional modification of other RNAs	17	1	A subset of the *ncRNA* biotype
rRNA	The RNA component of a ribosome	1	1	A subset of the *ncRNA* biotype
scaRNA	Non-coding RNA predicted using sequences from Rfam and miRBase	3	0	
TR C gene	Constant chain T cell receptor gene that undergoes somatic recombination before transcription	2	0	A subset of the *TR gene* biotype
TR V gene	Variable chain T cell receptor gene that undergoes somatic recombination before transcription	3	0	A subset of the *TR gene* biotype

1: sources – Ensembl [15], GenCode [16] and Vega [17]

Supplementary figures

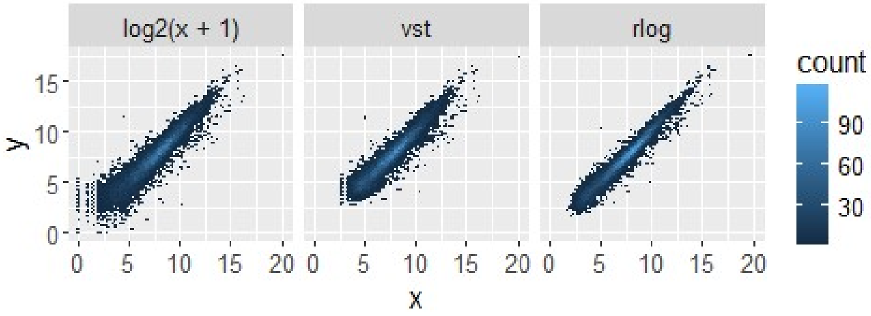

Figure S1 The effect of the different transformations on the raw read counts in the first two European dogs (CL_d1 and CL_d2).

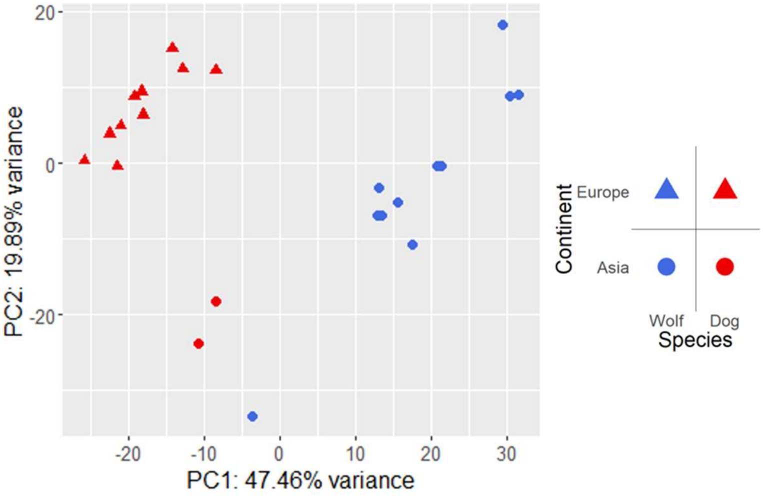

Figure S2 Principal component analysis of the raw read counts after regularized-logarithm transformation (first two PCs are shown). The amount of variance explained by the first two components is also indicated. Marker shapes indicate the origin of the samples, marker colors indicate the species.

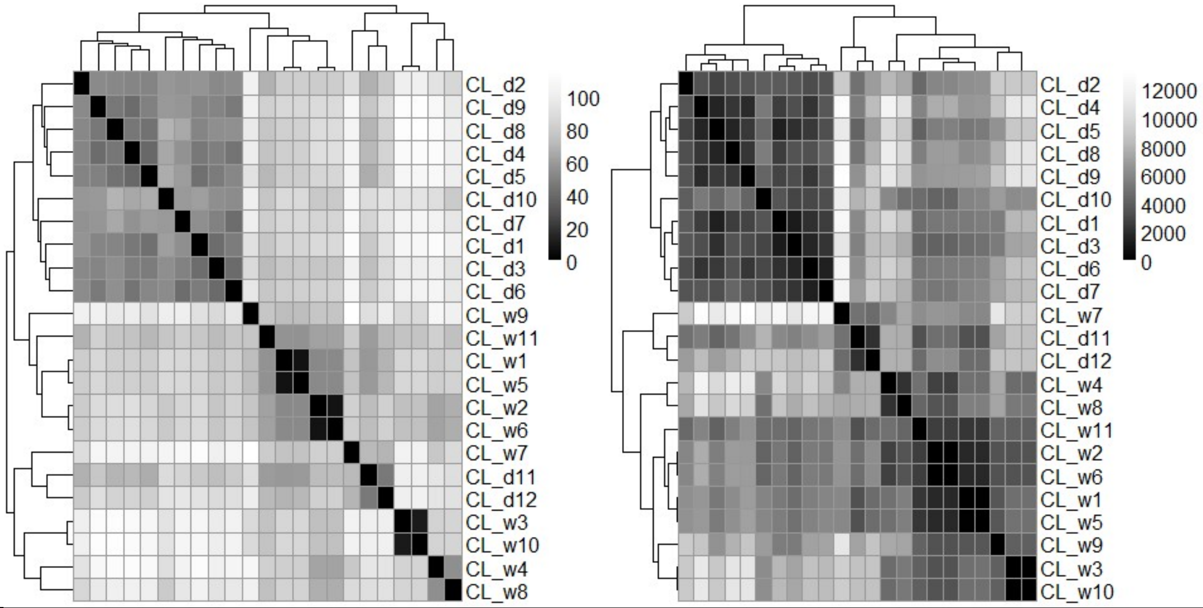

Figure S3 Euclidean (left) and Poisson (right) distances between the samples based on the read counts after regularized-logarithm transformation. Clustering of the test subjects is also shown at the margins of the figures.

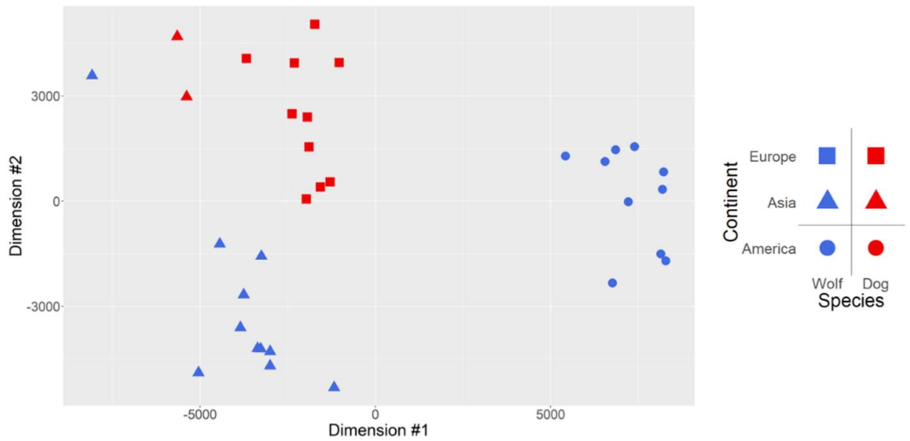

Figure S4 Multidimensional scaling of 33 canids from three continents: Europe (n=10) [12], Asia (n=13) [11; 13] and the Americas (n=10) [14].

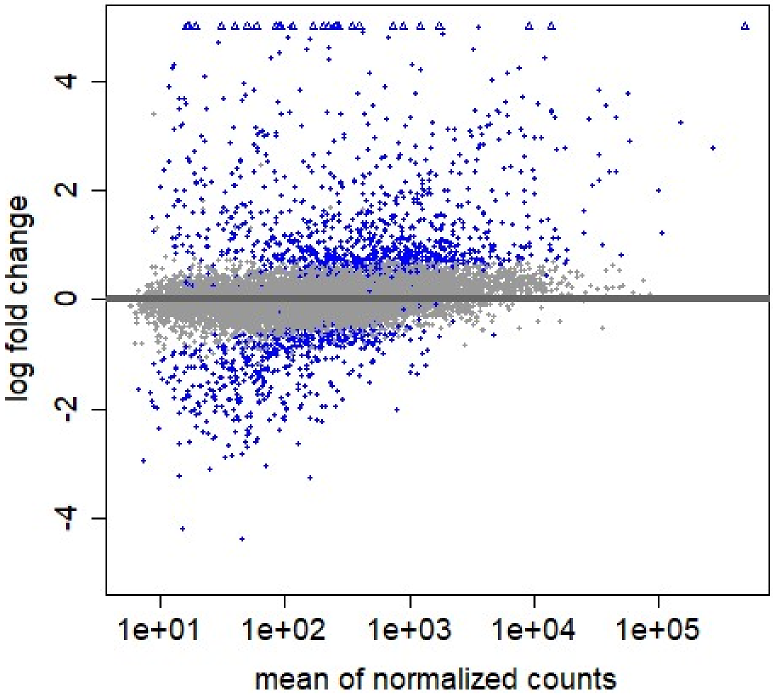

Figure S5 An MA-plot showing the mean normalized read counts of each gene (“A”; x axis) vs $\log_2$ fold change (“M”; y axis). Significant genes (n=1576) are highlighted in blue. Genes with a higher than 5, or lower than −5 $\log_2$ fold change value are indicated with triangles.

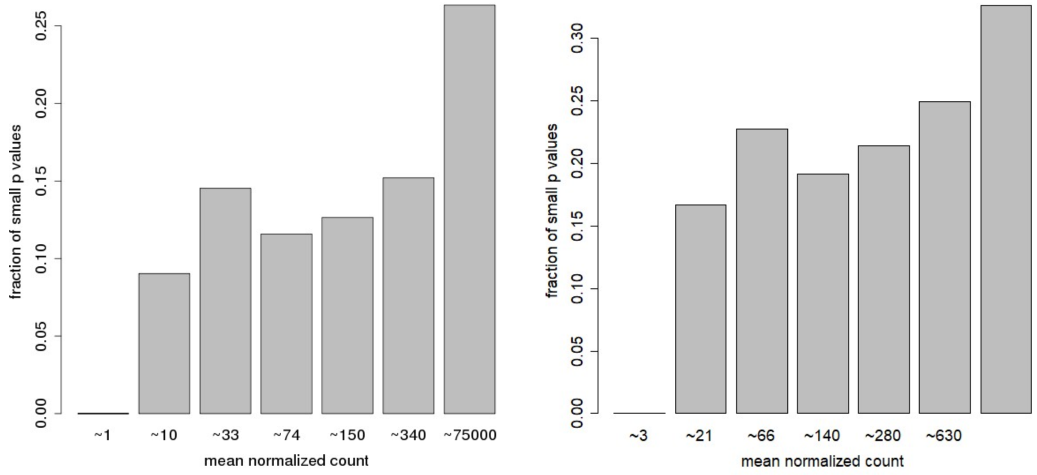

Figure S6 Proportion of low p-values ($p < 0.05$) among the tested genes divided into six quantiles, each containing 16.67% of the genes. Genes were divided into quantiles based on their normalized read count. The same plot is shown for the re-analysis of the data published by Yang et al. [15] (left) and for the analysis including our additional ten pet border collie dog samples (right).
